# Does Sleep *Really* Influence Face Recognition Memory?

**DOI:** 10.1371/journal.pone.0005496

**Published:** 2009-05-08

**Authors:** Bhavin R. Sheth, Ngan Nguyen, Davit Janvelyan

**Affiliations:** 1 Department of Electrical and Computer Engineering, University of Houston, Houston, Texas, United States of America; 2 Center for NeuroEngineering and Cognitive Science, University of Houston, Houston, Texas, United States of America; 3 California Institute of Technology, Pasadena, California, United States of America; National Institute of Mental Health, United States of America

## Abstract

Mounting evidence implicates sleep in the consolidation of various kinds of memories. We investigated the effect of sleep on memory for face identity, a declarative form of memory that is indispensable for nearly all social interaction. In the *acquisition phase*, observers viewed faces that they were required to remember over a variable retention period (0–36 hours). In the *test phase*, observers viewed intermixed old and new faces and judged seeing each before. Participants were classified according to acquisition and test times into seven groups. Memory strength (d′) and response bias (c) were evaluated. Substantial time spent awake (12 hours or more) during the retention period impaired face recognition memory evaluated at test, whereas sleep *per se* during the retention period did little to enhance the memory. Wakefulness during retention also led to a tightening of the decision criterion. Our findings suggest that sleep passively and transiently shelters face recognition memory from waking interference (exposure) but does not actively aid in its long-term consolidation.

## Introduction

Recently, evidence has emerged that implicates sleep in the consolidation of learning in memory. Sleep consolidates motor [1]–[4] and visual skill [5]–[9] learning, and declarative memories such as those for word associations [10].

Face recognition memory is a form of declarative memory that, like other declarative memories, is critically dependent on the medial temporal lobe of the brain [11]. Our memory for faces is crucial: We interact daily with a handful of familiar people, but passively see and remember the faces of many more people whom we casually encounter. Knowing that sleep helps consolidate myriad forms of learning including verbal sub-forms of declarative memory, and forming and retaining memories of old faces is vital for social interaction, one would expect sleep to enhance face recognition memory as well. There are at least two hypothetical ways by which sleep can operate on a declarative memory—sleep could either temporarily shelter the memory from exposure or interference during wakefulness, or consolidate the face memory so that it remains stable over a lasting period of time [12].

Both hypotheses, while they differ in important ways, are nonetheless in accord with the widespread belief that sleep is beneficial to the organism's fitness. Crick and Mitchison [13] theorized that sleep eliminates memories for irrelevant, or potentially harmful, items. On a task involving motor skill learning, Kuriyama et al. [14] claimed that sleep can selectively improve overnight performance on memories that need the greatest improvement, although more recent studies have cast doubt on this result [15]. On the basis of the studies cited above, one expects that sleep would also *selectively* help consolidate memories for behaviorally significant faces. Our study examined the influence of intervening sleep, intervening wake, and time of day on the retention and selectivity of face recognition memory.

## Methods

The experiment consisted of two parts: an acquisition phase, and a test phase.

### Stimuli

**Figure 1:**
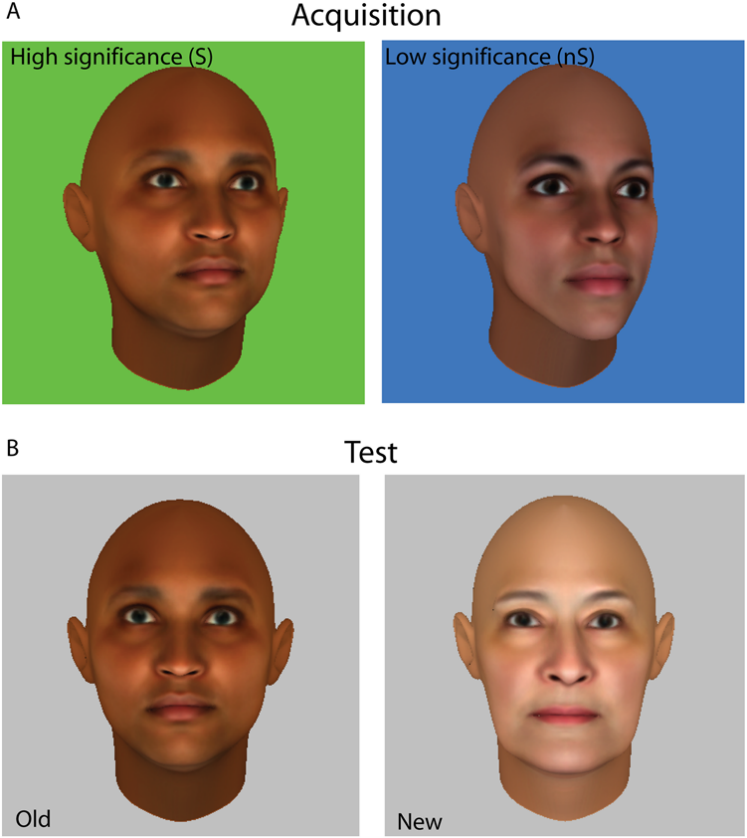
The stimuli used in the face recognition experiment. Stimuli were computer-generated faces, and had no distinguishing features, or hair on the face or head. A) Acquisition phase – Faces were shown on a homogenous green (significant or S faces) or blue (less significant or nS faces) background. The faces were shown in a 45° profile view. B) Test phase – Faces were shown on a uniform gray background. A test face could either be one of the faces shown earlier during acquisition (left) or a new face that the participant never saw before (right). An old face was equally likely to be an S face, as shown here, or an nS face. All test faces were shown in frontal view.

For each study participant, the stimuli were 60 faces (30 male, 30 female, various races represented) randomly generated by a software package (FaceGenModeller 2.2) Participants viewed the same set of 60 faces. The faces had no dermatological features, and no hair on the head or face (Fig. 1). This was done so that the participant could not ‘cheat’, i.e. recognize a face on the basis of some isolated feature unique to it (e.g. a mole on the left cheek distinguishes Cindy Crawford's face from that of others). It is notable that this treatment of the face stimuli used in the experiment rendered face recognition somewhat more difficult than usual (This is reflected in the relatively low d's in our cohort). Software for data acquisition and analysis was scripted in MATLAB (Mathworks, Inc.).

### Participants

All potential participants completed a screening questionnaire prior to selection. Individuals taking prescription, psychoactive medication or illicit drugs were excluded prior to randomization. Participants with known sleep disorders or abnormal sleep patterns, such as habitual sleep onset after 2 a.m., sleep duration less then 6 hr, or pathologic sleepiness (defined by an Epworth Sleepiness Scale score >10) were excluded.

**Figure 2:**
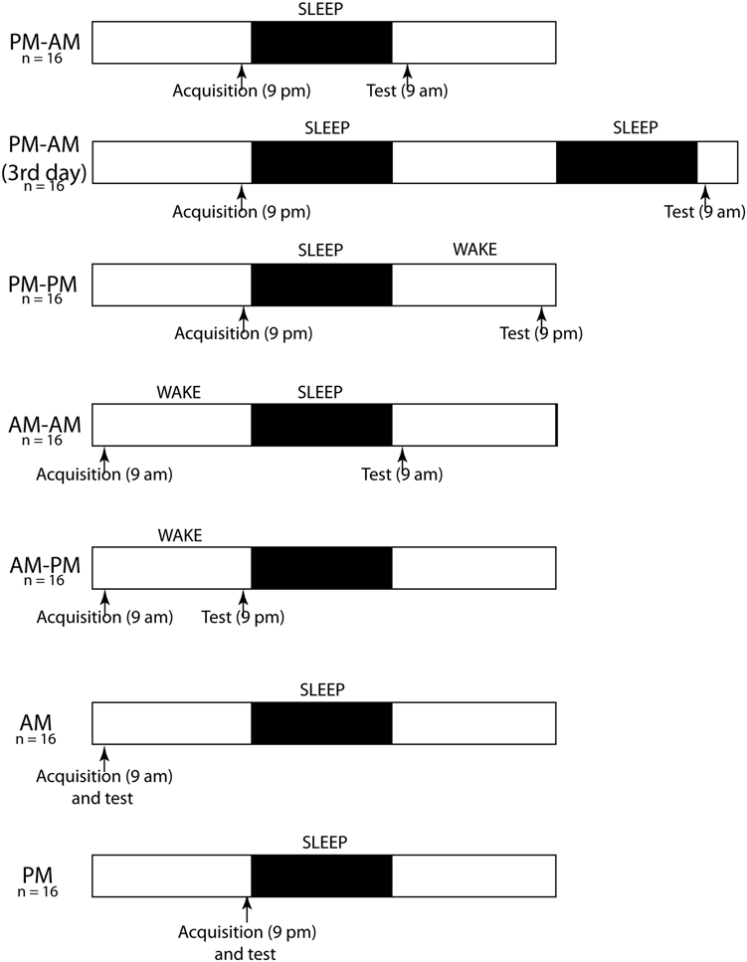
The experimental protocol. One hundred and twelve subjects were distributed into seven experimental groups [PM–AM, PM–AM(3^rd^ day), PM–PM, AM–AM, AM–PM, AM, PM] of 16 participants each. Acquisition and recognition test times for each group are illustrated with vertical arrows (see Methods for details).

One-hundred and twelve volunteers (mean age = 25 years, 3 months; 55/112 were female) enrolled and successfully completed the study. They were divided into seven groups of 16 participants each (Fig. 2): A) PM–AM: Participants in this group first viewed the faces in the evening (∼9 pm) and were tested on them approximately 12 hours later the following morning (2^nd^ day). The intervening retention period include a night of sleep. B) PM–AM (3^rd^ day): Participants in this group first acquired the faces in the evening (∼9 pm) of the first day and were tested on them approximately 36 hours following the initial acquisition on the morning of the 3^rd^ day. The retention period thus included two nights of sleep (compare with the group PM–AM above). C) PM–PM: Participants in this group acquired the faces in the evening (∼9 pm) and were tested 24 hours later (2^nd^ day, ∼9 pm). D) AM–AM: Participants in this group acquired the faces in the morning (∼9 am) and were tested 24 hours later (2^nd^ day, ∼9 am). E) AM–PM: Participants in this group acquired the faces in the morning (∼9 am) and were tested on them the same evening (1^st^ day, ∼9 pm). F) AM: Participants in this group acquired the faces in the morning (∼9 am) and were tested on them the same morning within five minutes following the initial acquisition (1^st^ day, ∼9 am). G) PM: Participants in this group acquired the faces in the evening (∼9 pm) and were tested on them the same evening within five minutes following the initial acquisition (1^st^ day, ∼9 pm). There are various ways of categorizing the groups. Groups A-E experienced a substantial retention period (12 hours or more), whereas groups F and G were tested almost immediately following acquisition. Amongst groups A-E, the first four groups (A–D) had at least one night of sleep prior to test, whereas group E remained awake during the retention period. Groups A–C got to sleep almost immediately following acquisition, whereas group D slept after ∼12 hours of being awake following acquisition.

Groups did not differ statistically in age (ANOVA: *F*(6,105) = 1.68, MS_e_ = 59.919, ns) or sex ratio (Kruskal-Wallis ANOVA: *χ*(6,105) = 4.18, MS_e_ = 804.5, ns). Participants in the PM–AM, PM–PM, AM–AM, and PM–AM(3^rd^ day) groups had a normal night of sleep (∼7.5 hours) immediately following acquisition, and, in the case of the PM–AM (3^rd^ day) group, a normal night of sleep on the second night following acquisition (7.9 hours). Sleep duration was monitored with sleep diaries; this was verified by actigraphy (Actiwatch, MiniMitter Inc.) on a limited number of participants.

Participants were not monetarily compensated for their participation, but were informed beforehand that the highest three test scores would be awarded cash prizes (1^st^ prize – $50, 2^nd^ prize – $30, 3^rd^ prize – $20). Thus, subjects knew there was a clear benefit to maximizing their point total. No stimulants, specifically alcohol, caffeine and tobacco, were permitted beginning from the night before acquisition until testing was complete (we had no independent means of verifying that they actually observed this restriction). The study was conducted with the understanding and written consent of each participant and under a protocol approved by the University of Houston Committee for Protection of Human Subjects.

### Procedure

The experimental procedure consisted of two phases—acquisition and test. The phases were separated in time by 12, 24 or 36 hours depending on group.

#### Acquisition phase

Participants sat comfortably in a chair in a well-lit room, and passively acquired the faces on a computer screen, one at a time for 2 s each with 2 s long intervening gaps. Faces were shown in 45° profile view (Fig. 1A). Faces were categorized into two classes: i) ‘highly significant’ or S, and ii) ‘less significant’ or nS. The face classes were easily distinguished by background color (Fig. 1A) *and* by a point score clearly written at the bottom right of the screen (not shown). S faces were presented on a uniform green background (Fig. 1A, left) and remembering each face correctly at test was worth 20 points; nS faces were shown on a uniform blue background (Fig. 1A, right) and remembering each face correctly at test was worth 1 point. Participants were informed of this distinction beforehand and our procedure ensured that participants were always aware to which category the face being presented belonged. Pilot studies indicated that background color did not affect memory for face recognition. There were 30 faces in each category. Face classes were randomly intermixed during acquisition. The entire set of computer-generated faces was shown five times with no interruptions (20 min. total). Participants were visually monitored to ensure they viewed each face; all participants viewed each face shown for the duration that it remained on the screen.

#### Test phase

A total of 60 faces in frontal view were shown. Thirty faces were old faces seen earlier during acquisition. Of those, one half (15) were S faces, one half were nS faces. The remaining thirty faces were new faces that participants had never seen before. Faces were presented one by one in random order on a uniform gray background (Fig. 1B); therefore, there were no clues about face class (old or new, S or nS). The participant had to make two binary responses for a given face—i) a “yes” or “no” response regarding whether the face was acquired earlier, and ii) a confidence rating of their initial response. Intermixing faces from the S and nS face classes minimized the possibility that participants could use different response criteria for different face classes (Wixted, 1992; Wixted & Stretch, 2000).

Participants were informed in the acquisition phase, then reminded prior to the test how test performance would be scored: A hit, i.e. a “yes” response at test, (i.e. “Yes, I have seen the face before”) on an S face, was worth 20 points, a “no” response (miss) was worth zero points. A “yes” response on an nS face was worth 1 point. A false positive, i.e. a “yes” response on a new face, was worth −10.5 points; a “no” response (correct reject) was worth 0 points.

### Data Analysis and Statistics

Memory (d′) and response bias (c) were calculated using standard measures (Macmillan & Creeman, 2005). Data from S and nS classes were combined for both measures. We measured recognition memory for each face class separately as well (**Results**).

A multiple linear regression model was applied to the complete set of d′ data. In the model, Y = X * B where Y represents the d′ data across all participants in our study, X represents the matrix of predictors corresponding to the variables found to be significant from our analysis, and B the vector of regression coefficients that provides the optimal least squares fit.

One-way and two-way ANOVAs were used to assess statistical significance. Significance is a p-value of 0.05 or less.

## Results

### Effect of stimulus significance

**Figure 3:**
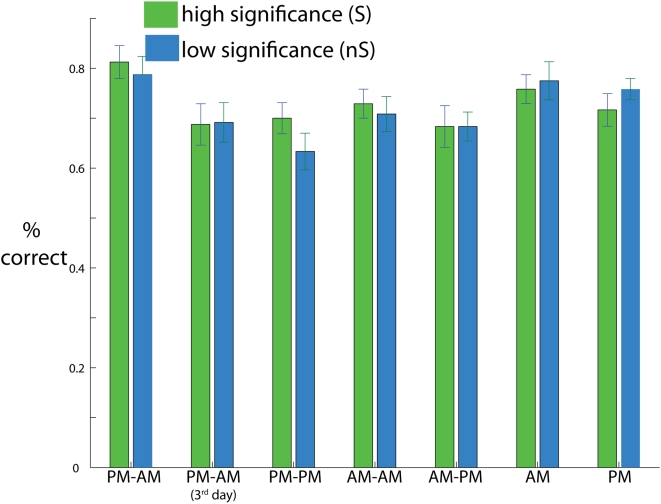
Recognition memory for highly significant (S) versus less significant (nS) faces. Group mean hit rates for highly significant (green bars) and less significant (blue bars) faces (ordinate) are plotted for each individual group (abscissa). Error bars are one s.e.m.

Faces were specified to be of low (nS) or high (S) significance (see **Methods**). A differential effect of stimulus significance at the encoding, consolidation, or retrieval stage of the memory process is likely to be reflected in the hit rate distributions at testing. However, no effect of stimulus significance was observed in the present study. Hit rates for each face class across all seven participant groups combined were not significantly higher for the S faces than the nS faces (*F*(6,210) = 0.14, ns), nor was there a statistically significant effect of stimulus significance for any individual group (Fig. 3). Furthermore, no significant interaction between face class and group was observed either (*F*(6,210) = 0.48, ns). In short, neither sleep nor the passage of time was found to have a significant effect on the selectivity of face recognition memory. On the basis of the fact that our manipulation of stimulus significance had no effect on memory, we combined results for both classes of stimuli – that is to say, face class is ignored henceforth.

First, we compared the performances of the groups of subjects who were required to retain memory over some period of time (12 hours or more), i.e. groups A–E, to see if sleep during the retention period had any beneficial effect on memory strength measured at test.

### Recognition memory strength (d′)

**Figure 4:**
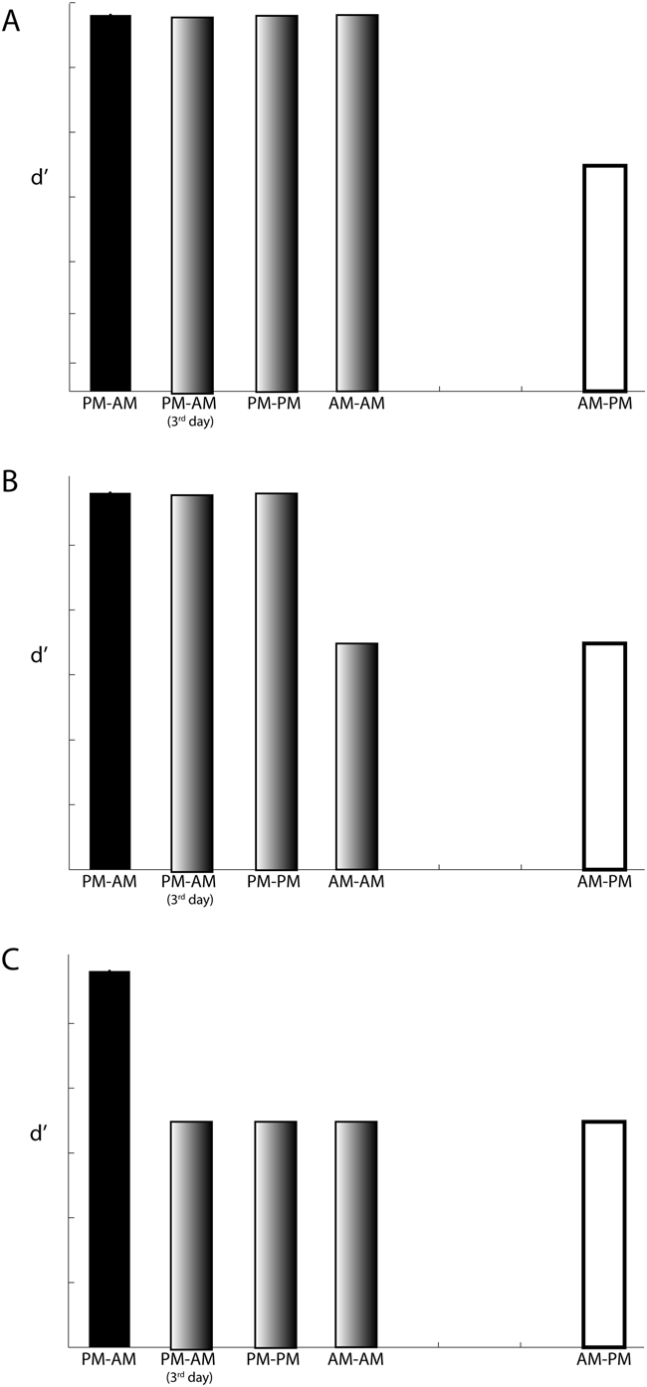
Three hypothetical scenarios of test performance (d′). Groups that sleep for the majority of the retention period are illustrated by black solid bars, groups that remain awake throughout retention are coded white, and groups that experience time in sleep as well as wake during retention are coded gray. (A) A hypothetical scenario in which a night of sleep during the retention period increases d's is shown. (B) A hypothetical scenario in which a night of sleep immediately following acquisition increases d's is shown. (C) (B) A hypothetical scenario in which a substantial time spent awake (12 hours or more) during the retention period leads to lower d's is shown.

It is important to point out that multiple factors interact within and between the various groups that need to be teased apart. In particular, there are three factors that we will focus on here: sleep during retention, sleep immediately following acquisition, and wakefulness during retention. From studies of other forms of memory, one posits that sleep some time after acquisition and before test is likely to be critical for the enhanced consolidation of memory for face identity. Once the memory is consolidated during sleep, there will be little deterioration. In the present study, the PM–AM, PM–AM (3^rd^ day), PM–PM, and AM–AM groups slept at least one night between acquisition and test; in comparison, the AM–PM group remained awake throughout the retention period. Fig. 4A shows a hypothetical scenario in which sleep during retention is the key variable driving test performance, leading to memory consolidation. The second factor is a variant of the first, viz. sleep immediately following acquisition. The argument is that memory is most susceptible immediately after acquisition, and sleep will be most effective in consolidating the memory if sleep immediately follows acquisition. Participants from the AM–AM group slept, but not right away following acquisition whereas those from the PM–AM, PM–PM and PM–AM (3^rd^ day) groups slept right after acquisition. Fig. 4B shows a hypothetical scenario in which sleep immediately following acquisition is the key variable benefiting memory consolidation. A third factor to consider is intervening wake. The idea is that when one remains awake, face recognition memory is rendered vulnerable to interference from faces (or from other external visual stimuli) one commonly encounters during the day. In the present context, the experimental group PM–AM spent little time in the wake state during the retention period. All the remaining groups spent a substantial time (12 hours or more) awake. Fig. 4C shows a hypothetical scenario in which intervening wake is detrimental to performance.

**Figure 5:**
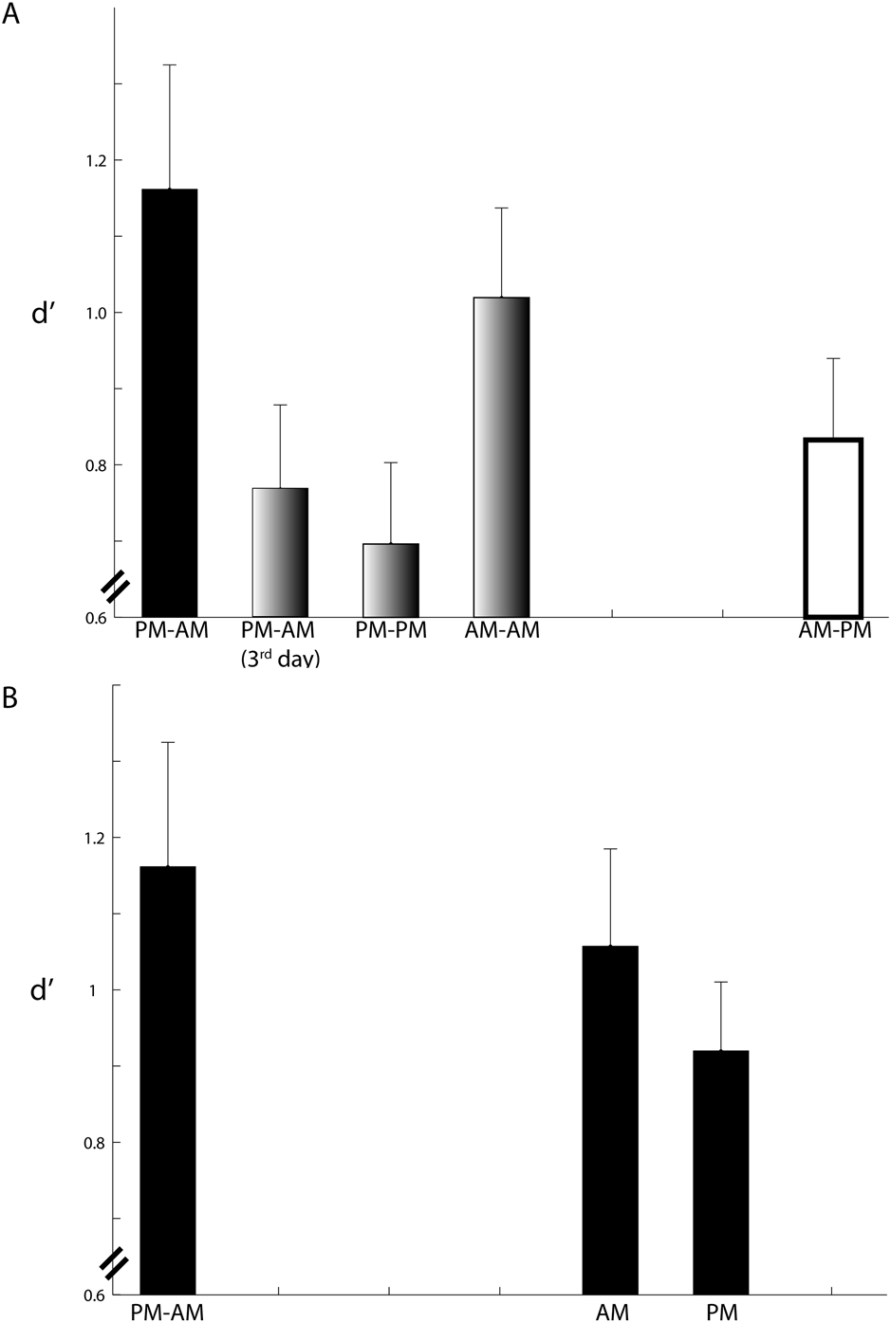
Recognition memory strength (d′). Memory for both classes of faces (S and nS) are combined (see Results for justification). Error bars are one s.e.m. (A) Mean±s.e.m. d's of the five groups that had to retain memory over a period of 12 hours duration or greater. (B) Mean±s.e.m. d's of the groups (AM and PM) that were tested immediately following acquisition with no retention period in between. d's of the PM–AM group are shown again for convenience.

Fig. 5A shows the d's of the five groups that had to retain the faces over some duration. The difference in d′ among the five groups who had to retain memory for some duration (12 hours or more) was marginally significant (*F*(4,75) = 2.42, MS_e_ = 0.242, p = 0.056), with the largest pairwise difference in d′ between the PM–AM and PM–PM groups (Fig. 5A). A visual comparison with the models illustrated in Fig. 4 shows that the data do not conform with the idea that sleep during the retention period enhances test performance (Figs. 4A, B vs. Fig. 5A). Rather, the data appear to be most in line—thought not entirely—with the idea that time spent awake during the retention period impairs test performance (Fig. 4C vs. Fig. 5A).

We examined the above three factors (sleep, sleep after acquisition, wake) more systematically in a multiple linear regression model. The value of a particular predictor varied in a binary fashion (0/1) depending on the group. For example, the predictor variable sleep was 1 for members of the group PM–AM, and 0 for members of the group AM–PM. Fitting the model to the d′ data for each subject from one of the five groups resulted in the following equation
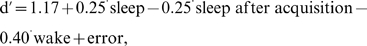
As indicated by the negative value (−0.40) of the coefficient of the wake predictor, intervening wakefulness of 12 hours or more had a detrimental impact on test d's. The sign of the coefficient was stable, as the 95% confidence interval of the coefficient was [−0.70, −0.10]. Although sleep during the retention period had a positive impact on test d's as indicated by the positive value of the coefficient of the sleep predictor, the corresponding 95% confidence interval [−0.10, 0.60] suggests that the effect of sleep is not siginficant. Sleep immediately following acquisition did not appear to have a positive impact on test d's, as indicated by the negative value of its corresponding coefficient. The model's overall fit was significant (*F* = 3.03, p = 0.034); however, the fraction of variance accounted for by the model (R^2^) was a mere 11%. The residual standard deviation, which is a measure of the average distance each observed d′ falls from its prediction from the model was 0.24; this informs us that the model predicted d's to a rather low level of precision. The results suggest that sleep during retention actively contributed little to the strength of face recognition memory. Rather, there was a small but significant negative contribution of intervening wake to memory strength. Of importance, there may be hitherto unknown factors and/or random noise in the d′ data unaccounted for by the model.

A second way of looking at the active contribution of sleep to the consolidation of face recognition memory is by examining whether sleep following acquisition improves or enhances test d's as compared to tests conducted right after acquisition. To this end, we compared the performances of the PM–AM group of participants who slept immediately after acquisition and for the majority of the retention period with two groups of subjects, AM and PM, who were tested on the faces almost immediately after acquiring them. d's of the PM–AM group were slightly higher than those of the AM and PM groups (Fig. 5B), but the difference was not statistically significant (*F*(4,45) = 0.86, MS_e_ = 0.272, p = 0.429). Thus, we did not find a significant measurable effect of sleep in enhancing performance beyond that measured at acquisition (see [4], [7], [15] for examples of sleep-dependent memory enhancement of other forms of memory).

### Response bias (c)

**Figure 6:**
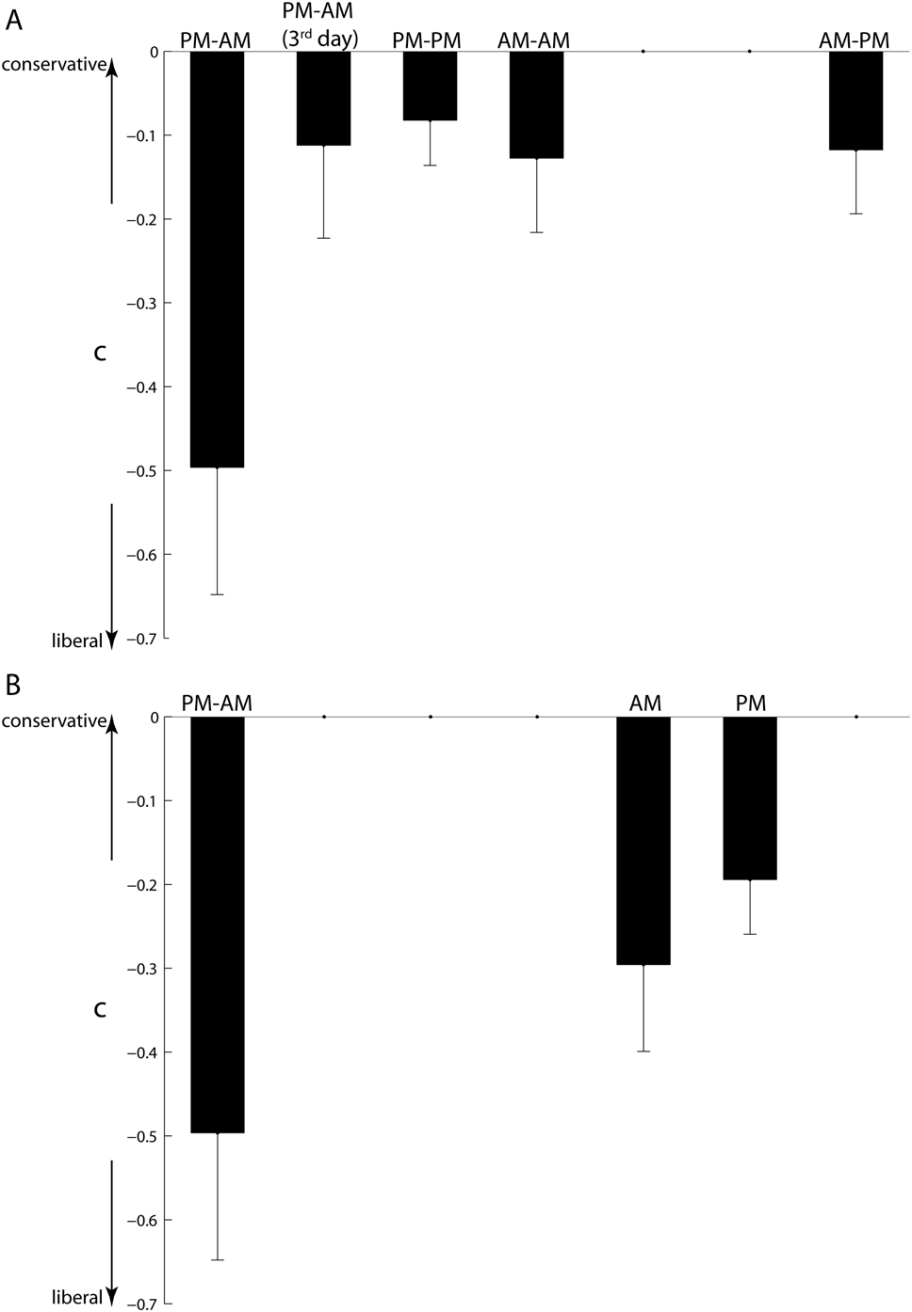
Response bias (c). (A) Mean±s.e.m. cs of the five groups that had to retain memory over 12 hours or more are shown. (B) Mean±s.e.m. cs of the AM and PM groups are shown. cs of the PM–AM group are shown again for convenience.

There was a significant difference in response bias (Fig. 6) across the five experimental groups that had to retain the memory (*F*(4,75) = 2.91, MS_e_ = 0.166, p = 0.027) as well as, more generally, across all seven experimental groups studied (*F*(6,105) = 2.28, MS_e_ = 0.153, p = 0.042). *Post-hoc* Tukey HSD tests showed that the PM–AM group (−0.50±0.15) was significantly more likely to claim having seen a test face (old or new regardless) before than the PM–PM group (−0.11±0.11). None of the other pairwise differences approached statistical significance. In this context, it is interesting to note that participants from the PM–AM group had the highest hit rates *and* the highest false alarm rates amongst all seven groups tested (Table 1); moreover, the mean values of c for the AM and PM conditions were comparable to that on the PM–AM condition (Fig. 6B) and numerically more negative than that of other groups, which is consistent with the idea that if participants are not awake for a substantial duration between acquisition and test (PM–AM, AM, and PM), their decision-making is more liberal.

**Table 1:** Hit rates (HRs) and false alarm rates (FARs).

Participant group	HR	FAR
	mean±s.e.m.	mean±s.e.m.
PM–AM	0.80±0.03	0.47±0.04
PM–AM (3^rd^ day)	0.69±0.04	0.40±0.04
PM–PM	0.67±0.03	0.39±0.03
AM–AM	0.72±0.03	0.37±0.04
AM–PM	0.68±0.03	0.40±0.03
AM	0.77±0.02	0.44±0.05
PM	0.74±0.03	0.40±0.03

As before, we examined the effect of the same three factors (sleep, sleep after acquisition, wake) on response bias (c) in a multiple linear regression model. Fitting the predictors to the c data resulted in the following equation
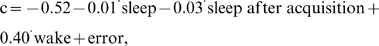
Here again, the clear effect on response bias is of intervening wake. As indicated by the positive value (+0.40) of the coefficient of the wake predictor, intervening wakefulness of 12 hours or more rendered response bias more conservative. The 95% confidence interval of the coefficient was [+0.15, +0.65], indicating that the predictor had a stable and significant effect on bias. The other two predictors – sleep during retention and sleep after acquisition – had minimal effect on response bias as indicated by the near-zero values of their corresponding coefficients and the 95% confidence intervals of their respective coefficients (sleep – [−0.30, +0.28]; sleep after acquisition – [−0.22, +0.28]). Overall, the model's fit was significant (*F* = 3.91, p = 0.012); again, the fraction of variance accounted for by the model (R^2^) was a mere 13%. The residual standard deviation was 0.16. Overall, the results suggest that intervening wake during retention rendered the subject less likely to report seeing a test face before. Also, other factors need to be considered to improve the quality of the fit.

### Subjective alertness

Level of alertness was reported on the seven point (1 is most alert) Stanford Sleepiness Scale [16], [17] at acquisition and again upon testing. Reported scores on the scale did not vary significantly across group at acquisition (*F*(6, 105) = 1.30, MS_e_ = 2.40, ns; Table 2) or at test (*F*(6, 105) = 1.83, MS_e_ = 4.07, ns; Table 2).

**Table 2:** Stanford Sleepiness Scale scores.

Participant Group	Acquisition	Test
	mean±s.e.m.	mean±s.e.m.
PM–AM	2.7±0.4	1.6±0.2
AM–PM	1.9±0.2	2.5±0.5
PM–PM	2.9±0.4	2.3±0.4
AM–AM	2.9±0.2	2.4±0.3
AM	2.9±0.3	2.9±0.3
PM	2.8±0.3	3.1±0.4
PM–AM (3^rd^ day)	2.3±0.3	2.3±0.4

A related question is whether there was a relationship between subjective alertness and recognition memory at test for the variables that were found to be significant predictors of performance at test above, namely wakefulness during the retention period, and time of testing. We did not find a significant difference in the reported alertness scores at acquisition or test between the participants that remained awake for 12 hours or more during the retention period and the participants that remained awake for 4 hours or less. The reported scores at acquisition and test between participants who ran the memory test in the morning versus in the evening were not significantly different either. Thus, differences in subjective alertness scores at acquisition or test did not parallel differences in memory strength at test.

That self-reports of alertness are not predictors of performance is further highlighted by the fact that, measured across the entire cohort, the correlations between d′ on the one hand and reported alertness scores at acquisition and at test on the other were not significant.

## Discussion

Our study of face recognition memory did not yield the result that sleep per se enhanced memory strength. Time spent in sleep during the retention period had little effect on face recognition memory, as did sleep immediately following acquisition. On the other hand, fitting the data with a multiple linear regression model indicated that time spent awake (12 hours or more) over the retention period modestly but significantly reduced memory at test and rendered participants more conservative at test, i.e. less likely to feel familiar with a test face. A plausible interpretation of our findings is that in wakefulness, ongoing sensory stimulation interferes with the visual memory; sleep, by sheltering the visual memory from sensory interference, temporarily prevents memory loss, but subsequent wake washes out the effects of sleep, with the result that sleep has no long-lasting impact on retention of face recognition memory.

There are at least two possibilities as to what comprises sensory interference. Interference could be from other, more faces or from external visual stimuli in general. One typically sees or visualizes far fewer faces in sleep than while awake. Viewing a lot of faces could corrupt one's memory of faces seen earlier. This is tantamount to interference between memorized and perceived faces in brain areas or circuits where face identity is processed and remembered, such as the fusiform face area in humans [18], [19]. Alternatively, visual stimulation while awake might corrupt all forms of visual memory including those for face identity. This is tantamount to interference between memorized and perceived faces in brain areas or circuits prior to face processing, perhaps involving interactions between spatial filters in some low-level visual cortical area [20].

The present findings appear to contradict the conclusions of an earlier study on the role of sleep in face recognition memory [21], however we contend that when analyzed more carefully, our study can account for the earlier claim. Subjects in the earlier study viewed faces in the evening, then either slept normally on the night following (sleep condition), or, on a different night two weeks apart, remained awake (wake condition) overnight during which there were no restrictions on their visual exposure. Recognition testing took place on the second evening after learning. The authors reported finding that sleep after learning, as compared to wakefulness, moderately enhanced recognition memory. These findings, as Wagner et al. themselves admit, are “consistent with either view [improved memory consolidation or reduced forgetting in sleep] and therefore do not contribute to solving the fundamental issue of the mechanisms of sleep-associated consolidation” (pp. 684 of [21]). On the basis of our report, which is more thorough insomuch as it studies many more groups or conditions and examines multiple different factors influencing face recognition memory, we offer a different, arguably simpler interpretation of their results based on the following. Participants in the earlier study experienced 7–8 more hours of visual interference in the wake condition than in the sleep condition; wakefulness during retention, as our study suggests, diminishes memory retention. We believe this is a reasonable explanation for why subjects in [21] performed better on the sleep condition.

There are important caveats to our findings as well. First, performance was slightly, but not significantly, higher on the 12 hour PM–AM condition than on the 5 minute AM and PM conditions (Fig. 5B), which *seems* to mildly contradict the claim that the only benefit of sleep on memory for faces is in the removal of interference. Furthermore, one might argue that participants on the PM–AM condition presumably did see some other faces between acquisition and test (during the 4.5 hours that they were awake between acquisition and test), whereas participants on the AM and PM conditions presumably did not, except perhaps the experimenter's. This is not entirely true, however: Thirty new faces were shown at test, which occurred almost immediately after acquisition on the AM and PM conditions. It is likely that these new faces interfered with the nascent memory of the faces shown earlier at acquisition, causing the slight deterioration in test performance on the 5 min. conditions compared with the PM–AM condition. On this basis, we do not believe that the comparison between the 5 min. conditions and the PM–AM condition uncovers evidence in favor of a proactive role of sleep in face memory consolidation. Second, as mentioned above, participants in our PM–AM condition were awake for some time (4.5 hours or so); the time spent awake may have diminished performance to some extent, which is consistent with our assertion that wakefulness interferes with the retention and consolidation of face recognition memory. One way of examining this possibility is to run a “pure” sleep condition in which the participant spends no time awake and test is typically 7–8 hours after acquisition. Third, and as mentioned earlier, other factors e.g. time of day, are likely to play a role. On this note, the AM group performed better numerically than the PM group (compare their d's in Fig. 5B), although the difference was not significant. On a related note, the AM–AM group also performed slightly better than the PM–PM group (Fig. 5A). It would appear therefore that acquisition of faces and/or testing in the morning compared with in the evening could improve performance. On the other hand, the PM–AM (3^rd^ day) group who were tested in the morning did not perform as well, numerically, as the AM–PM group who were tested in the evening, which would mildly contradict the idea that testing in the morning (and, by proxy, a preceding night of sleep) is *the* critical factor benefitting performance. Nonetheless, time of testing could be *a* factor affecting d's. It bears mention that participants who were tested in the morning typically slept the night before. If future experiments bear out the benefit of morning testing and morning testing is found to be associated with prior sleep, it would imply that sleep improves test performance by temporarily enhancing attention, motivation, or brain restitution, not via some long-term process of memory consolidation. Finally, in our study, unfamiliar face recognition (i.e. recognition of pictures of faces not known to the individual) was studied, rather than familiar face recognition (i.e. recognition of people known to the individual). There are important distinctions between the two forms of face recognition [22], and it is possible that our findings do not generalize to the more common familiar face recognition.

### Sleep and memory selectivity

There has long been a tradition of speculation that sleep renders memory more selective [13]. From this view, sleep selectively enhances ‘stronger’ memories, or memories that are behaviorally or biologically relevant – perhaps via some sleep dependent mechanism such as replay in the hippocampus [23] – and/or impairs ‘weaker’ memories that are not relevant or are harmful – perhaps via long-term synaptic depression [24]. Behavioral tests of this idea have been few and far between, and the findings thus far are not conclusive. Kuriyama et al. [14] studied the effect of sleep on learning a sequence of typing movements, and claimed that the slowest transitions – the “problem-points” in the sequence most in need of improvement – were the ones that showed the greatest improvement in speed following sleep. A more recent study examined accuracy on the same task [15], and found that problem-points in the sequence remained even after sleep; only their identities changed. Thus, it was not conclusive that sleep had a selective effect on motor sequence learning.

The present study is inconclusive in this regard as well. The lack of differences in memory performance for high significance vs. low significance faces can not be attributed to a lack of modulation by sleep, as it was found for the 5 min AM and PM conditions as well (see Fig. 3). In fact, the significant/insignificant distinction appears not to have been meaningful to performance at all. One possibility could be that significance was determined by the experimenter here; it remains to be seen if sleep influences selectivity when the study participant, not the experimenter, gets to determine what is significant and what is not.

In conclusion, our study does not support the proposal that sleep improves the consolidation of face recognition memory [25], [26]. Rather, the study clearly suggests that substantial time spent awake diminishes the retention of face memory. Conversely, passive sheltering of the memory from interference enhances its retention. Sheltering from visual interference is not the exclusive purview of sleep [27], [28]: eye closure in wake is another means of achieving the same end. Future experimental studies must address what constitutes interference and how to shelter face memory from interference while awake.
